# Final irrigation protocols can be used to promote stable long-term bond strength of AH Plus to dentin

**DOI:** 10.1590/1678-7757-2023-0005

**Published:** 2023-05-26

**Authors:** Talita TARTARI, Caroline WICHNIESKI, Renato Menezes SILVA, Ariadne LETRA, Marco Antonio Hungaro DUARTE, Clovis Monteiro BRAMANTE

**Affiliations:** 1 Universidade Estadual de Campinas Faculdade de Odontologia de Piracicaba Departamento de Odontologia Restauradora Piracicaba SP Brasil Universidade Estadual de Campinas, Faculdade de Odontologia de Piracicaba, Departamento de Odontologia Restauradora, Piracicaba, SP, Brasil.; 2 Universidade de São Paulo Faculdade de Odontologia de Bauru Departamento de Dentística Operatória, Endodontia e Materiais Odontológicos Bauru SP Brasil Universidade de São Paulo, Faculdade de Odontologia de Bauru, Departamento de Dentística Operatória, Endodontia e Materiais Odontológicos, Bauru, SP, Brasil.; 3 Faculdade Herrero Curitiba PR Brasil Faculdade Herrero, Curso de Odontologia, Curitiba, PR, Brasil.; 4 Pontifícia Universidade Católica Departamento de Endodontia Curitiba PR Brasil Pontifícia Universidade Católica, Departamento de Endodontia, Curitiba, PR, Brasil.; 5 University of Pittsburgh School of Dental Medicine Department of Endodontics Pittsburgh PA USA University of Pittsburgh, School of Dental Medicine, Department of Endodontics, Pittsburgh, PA, USA.; 6 University of Pittsburgh School of Dental Medicine Department of Oral and Craniofacial Sciences Pittsburgh PA USA University of Pittsburgh, School of Dental Medicine, Department of Oral and Craniofacial Sciences, Pittsburgh, PA, USA.

**Keywords:** Resin cements, Root canal irrigants, Root canal obturation, Scanning electron microscopy, Time factors

## Abstract

Irrigation solutions might affect dentin surface characteristics and, consequently, endodontic sealers adhesion.

**Objective:**

This study analyzed the effect of different final irrigation protocols on push-out bond strength (BS) of AH Plus to dentin seven days and 20 months after obturation. Scanning electron micrographs were obtained from the dentin surface of one sample/group after final irrigation.

**Methodology:**

Canals of bovine incisors were instrumented and received final irrigation with (n=21): G1 – 2.5% sodium hypochlorite (NaOCl) + distilled water; G2 – 2.5% NaOCl + 17% EDTA; G3 – 2.5% NaOCl + 17% EDTA + 2.5% NaOCl; G4 – 2.5% NaOCl + 17% EDTA + 2% chlorhexidine (CHX); G5 – mixture 5% NaOCl + 18% etidronate (HEDP); and G6 – mixture 5% NaOCl + 10% tetrasodium EDTA (Na4EDTA). After irrigation, one root/group was split and images were obtained by scanning electron microscopy (SEM). The other 20 roots/group were filled with only AH Plus sealer. Three slices/root were used for push-out assessment seven days and 20 months after obturation. One-way analysis of variance and Tukey (α<0.05) were used to compare the results among experimental groups, and unpaired t-test (α<0.05) was used to compare the results of the same group over time.

**Results:**

The photomicrographs showed that, excepting G1, all groups completely removed the smear layer from the samples. In G2 and G4, the opening of the dentin tubules enlarged. In G3, erosion was observed in the peritubular and intertubular dentin. Values of the BS in the seven days were G2=G3=G4=G5>G6=G1 and in the 20 months were G3=G5>G6=G4>G1=G2. G3, G5, and G6 presented values of BS in 20 months similar to the values of seven days (P>0.05).

**Conclusions:**

The final irrigation protocols tested produced dentin surfaces with different characteristics. Only G3 and G5 presented high BS values that were stable over time.

## Introduction

Adhesion is a complex process involving molecular interactions at the interface of an adherend, which in Dentistry can be the dentin, with an adhesive, such as the endodontic sealers. It can be obtained by physical, chemical, and/or mechanical bonding between materials, being the latter the most effective in creating strong joints.^1^ In Endodontics, the adhesion of the root canal sealers to the dentin walls is important in static situations to eliminate spaces that allow for the percolation of fluids,^2^ prevent microbial reinfection, and trap remaining bacteria.^3^ On the other hand, in dynamic situations, this adhesion should avoid the displacement of the obturation during a subsequent handling.^4 , 5^ However, the achievement of a strong and long-term stable adhesion between the endodontic sealers and the root canal walls is still a current challenge.

The properties of the dentin surface are crucial to reach good interaction with the dental materials and, among the relevant features of this substrate are the cleanliness, roughness, and wettability.^1^ Studies have shown that irrigation solutions applied in the process of shaping and cleaning of the root canal system might alter the characteristics of dentin substrate^6 , 7^ and consequently interfere with the adhesion of the endodontic sealers.^8 - 11^ Sodium hypochlorite (NaOCl), the main irrigant in Endodontics , denatures the collagen of dentin^7^ and its oxidizing effect can compromise the polymerization of the resin sealers.^11 , 12^ The EDTA solutions used to remove the smear layer increase the roughness of the surface^6 , 13^ and produces a large demineralized dentin zone with great amounts of collagen fibrils exposed.^7^ These fibrils can contribute to the adhesion of endodontic sealers, such as AH Plus.^14^ However, an incomplete infiltration of the dental materials into the full extension of the exposed collagen matrix results in a weak bond interface that is degraded over time.^15 - 17^ This degradation is caused by the action of host-derived proteolytic enzymes called metalloproteinases on the denuded collagen fibrils.^15 , 17^

In theory, some irrigation protocols could help to overcome the problem of the adhesive interface degradation by providing stronger, favorable, and stable bonding of endodontic sealers over time. One of the protocols proposes the application of chlorhexidine (CHX) after the use of the decalcifying agent to inhibit the matrix metalloproteinases.^18^ Another option is to use weaker chelating agents, such as etidronate (HEDP) or tetrasodium EDTA (Na_4_EDTA), since they promote moderate demineralizing effects that supposedly allow for an adequate infiltration of the filling material throughout the depth of the exposed collagen matrix.^8^ Removing the exposed collagen fibrils with a deproteinizing agent, such as NaOCl, after the chelating agents is a third alternative.^19^

Also, the selection of an endodontic sealer is mandatory for the long-term success of root canal treatment and the sealing ability is one of the most important properties to be considered.^20^ When the sealer does not perform its function, a lack of adequate seal is implicated as a source of failure due to the movement of tissue fluids, bacteria, and their toxins into or out the canal space, by permeable spaces and voids in the filled canal.^21 , 22^ AH Plus is an epoxy resin-based sealer widely used and considered the gold standard for experiments assessing root canal obturation.^23^ The reasons for its use are the physical properties including adequate dimensional stability,^24^ low solubility and disintegration, and excellent adhesion to dentin, higher than other sealers.^25^

Due to a lack of studies evaluating long-term effects of different irrigation protocols on the dentin bond strength (BS) of endodontic sealers, this article presents the impact of different irrigation protocols in the BS of AH Plus to dentin in seven days and 20 months after the obturation. Moreover, scanning electron micrographs were obtained from one sample per group to verify the dentin surface. This study was designed to test if different irrigation protocols have a similar impact on the immediate and on the long-term BS of AH Plus sealer to dentin.

## Methodology

### Specimen preparation

This study was submitted and registered at the Animal Ethics Committee of the institution where the study was conducted under the number 016/2017.

Bovine incisors with completely formed roots were sectioned transversally at the cementoenamel junction and apically at the root end to obtain a root segment with approximately 14 mm of length. The cuts were performed using a diamond disc (KG Sorensen Ind. e Com., São Paulo, SP, Brazil), under water cooling. The apex portion of the canals was sealed with wax to keep the irrigants inside the root canal. Then, the canals were instrumented using the ProTaper Universal system (Dentsply Sirona, Ballaigues, Switzerland). The cervical third was prepared with the SX instrument and the other thirds with the instruments S1, S2, F1, F2, F3, F4, and F5.

The root canals were irrigated with 2 mL 2.5% NaOCl before the use of the SX file and at each instrument change. Irrigation was performed with a 10 mL disposable plastic syringe attached to a polypropylene capillary tip (Ultradent Products Inc., South Jordan, UT, USA). After the final irrigation, the root canals were completely dried with absorbent paper points (Dentsply, Petropolis, RJ, Brazil).

The roots were then distributed, with the aid of the Bioestat 5.0 program (Bioestat, Belém, PA, Brazil), into six groups (n=21) according to the final irrigation protocols: G1 – 2.5% NaOCl (5 min) + distilled water (1 min); G2 – 2.5% NaOCl (5 min) + 17% EDTA (1 min); G3 – 2.5% NaOCl (5 min) + 17% EDTA (1 min) + 2.5% NaOCl (1 min); G4 – 2.5% NaOCl (5 min) + 17% EDTA (1 min) + 2% CHX (2 min); G5 – Mixture 5% NaOCl + 18% HEBP (5 min); and G6 – Mixture 5% NaOCl + 10% Na_4_EDTA (5 min). For the irrigation procedure, 1 mL of irrigant was used per min of the time set per solution. After using each irrigant, the solutions were aspirated, and the canals were rinsed with 2 mL of distilled water for 1 min to remove the remnants of the irrigators. At the end, the canals were dried with absorbent paper points.

For the obturation of the canals, the AH Plus sealer (Dentsply DeTrey, Konstanz, Germany) was prepared according to the manufacturer’s instructions. To obtain a similar composition of the sealer in all groups, only the middle portion of the tubes was used.^26^ The root canals of 20 samples/group were filled using only the sealer to have exclusively the results of the BS between the sealer and the dentin and avoid confounding factors.^9 , 27^ AH Plus was inserted into canals with the aid of a lentulo spiral #40 (Dentsply/Maillefer) until the entire canal was filled. The roots were digitally radiographed in two angulations: mesiodistal and buccolingual directions. Those with voids or bubbles were discarded and replaced with new samples. The roots of all groups were stored at 37°C and 100% humidity, and on the day after the obturation, the wax on the apical portion of the roots was removed and the coronal and apical extremities of the canals were sealed with glass ionomer cement.

After one week, 10 roots of each group were sectioned in a precision cutting machine (Isomet, Buehler, Lake Bluff, IL, USA) under constant water cooling. To remove the glass ionomer cement from the extremities of the canal, a 1 mm slice was cut at the cervical and another at the apical portion of the roots and discarded. Then, cuts were performed with 2 mm of distance between each other, so six slices were obtained from each root (two per root third). The most cervical slice of each third was selected for the push-out test and the other one was discarded. The other 10 roots/group were stored for 20 months and then were cut in slices, in the same way.

Each slice was labelled. Thickness was confirmed with a digital caliper with 0.02 mm accuracy (Mitutoyo, Tokyo, Japan), and the apical and cervical diameters of the obturated area were measured with the stereomicroscope Stemi 2000 C (Carl Zeiss, Gottingen, Germany).

For the push-out assessment, the slices were placed on a base with a central hole. The tests were performed by applying a compressive load to the apical-coronal direction by using different cylindrical plunger tips. The selection of the plunger for each slice was based on the measures previously obtained by the stereomicroscopy. The biggest plugger, compatible with the obturation area in the apical surface of the slice, was used. The plunger tip was centred on the filling material without touching the surrounding dentin surface. Loading was performed on a universal testing machine (Instron 3342 – Instron Corporation, Canton, MA, USA) at a 0.5 mm/min speed until bond failure occurred.

The megapascals (MPa) value of push-out BS of each specimen was estimated by dividing the force required to dislodge the filling material (F in kN) by the bonding surface area of the root canal filling (mm^2^ ), using the equation σ=F/A. The bonding surface area of each sample was estimated by the equation of a conical frustum: A=π(R2+R1)×[h2+(R2-R1)^2^ ]^0^, where R1=base radius, R2=top radius, and h=height of the frustum.

One root of each group was not obturated and was used to obtain images of the dentin surface by scanning electron microscopy (SEM). The roots were split longitudinally and mounted on SEM stubs using a double-faced carbon tape, then they were sputter coated with platinum and palladium, and evaluated under the FEI Nova NanoSEM 230 ultra-high resolution scanning electron microscope (FEI Europe, Eindhoven, Netherlands). Photomicrographs were taken from the middle portion of the root fragments at 25000× magnification.

### Statistical analysis

The sample size was determined based on a pilot study in which the push-out BS mean was 15.9 ± 4.2 (± standard deviation). Thus, using the unpaired *t* -test with 80% power and a 5% bilateral a, to detect a 20% difference between the same group over time, the required sample size was 27 specimens per group, which means one slice/root third of nine teeth. Considering that samples could be lost during the experiment, 10 teeth were prepared per group for each period of analysis.

The data of push-out BS obtained showed a normal distribution in the preliminary normality test (P>0.05); thus, the one-way analysis of variance (ANOVA) and Tukey *post-hoc* tests were used to compare the results among the different irrigation protocols at the same experimental time. Unpaired *t* -test was used to compare the results of the same irrigation protocol seven days and 20 months after the obturation. The confidence level of all tests was set at 95%.

## Results

The effect of root canal thirds was assessed. However, no significant differences were found among all groups in both time periods (P>0.05).

Table 1 shows the mean and standard deviation of the dentin BS of the AH Plus sealer for each of the tested irrigation protocols seven days and 20 months after the obturation. One-way ANOVA showed that seven days after obturation, the values of the push-out BS of G2, G3, G4, and G5 were statistically similar (P>0.05) and higher than the other groups (P<0.01). G1 and G6 presented the lower values of the bond (P<0.01) and were similar to each other (P>0.05). Regarding the values obtained 20 months after the root canal fillings, G3 and G5 presented similar values of BS (P>0.05) and higher values from all other groups (P<0.01). They were followed by G4 and G6, which were similar (P>0.05). The lower bond values were associated with G1 and G2 (P<0.01), which were statistically similar (P>0.05).

**Table 1:** Mean (X) and standard deviation (SD) for the push-out bond strength (MPa) of the epoxy resin sealer AH Plus seven days and 20 months after the root canal filling according to the irrigation protocols employed

GROUPS	MPa	
	7 days* X±SD	20 months* X±SD
G1 – 2.5% NaOCl (5 min) + distilled water (1 min)^†^	15.6±3.9^Ba^	11.7±3.5^Cb^
G2 – 2.5% NaOCl (5 min) + 17% EDTA (1 min) ^†^	19.4±4.0^Aa^	10.4±4.6^Cb^
G3 – 2.5% NaOCl (5 min) + 17% EDTA (1 min) + 2.5% NaOCl (1 min) ^†^	18.8±3.5^Aa^	20.0±3.8^Aa^
G4 – 2.5% NaOCl (5 min) + 17% EDTA (1 min) + 2% CHX (2 min) ^†^	19.0±2.7^Aa^	15.3±3.4^Bb^
G5 – Mixture 5% NaOCl + 18% HEBP 18% (5 min) ^†^	19.7±2.6^Aa^	19.5±3.4^Aa^
G6 – Mixture 5% NaOCl + 10% Na4EDTA (5 min) ^†^	14.1±3.2^Ba^	15.7±4.5^Ba^

*One-Way ANOVA with Tukey post-hoc p-value<0.01; Different capital letters in columns indicate statistically significant intergroup differences in the same time period; ^†^Unpaired t-test p-value<0.05; Different lowercase letters in rows indicate statistically significant intragroup differences.

The comparison of the values of the same group over time by the unpaired *t* -test showed that only G3, G5, and G6 presented results of push-out BS in 20 months similar to the values obtained in the seven days (P>0.05).

**Figure 1:**
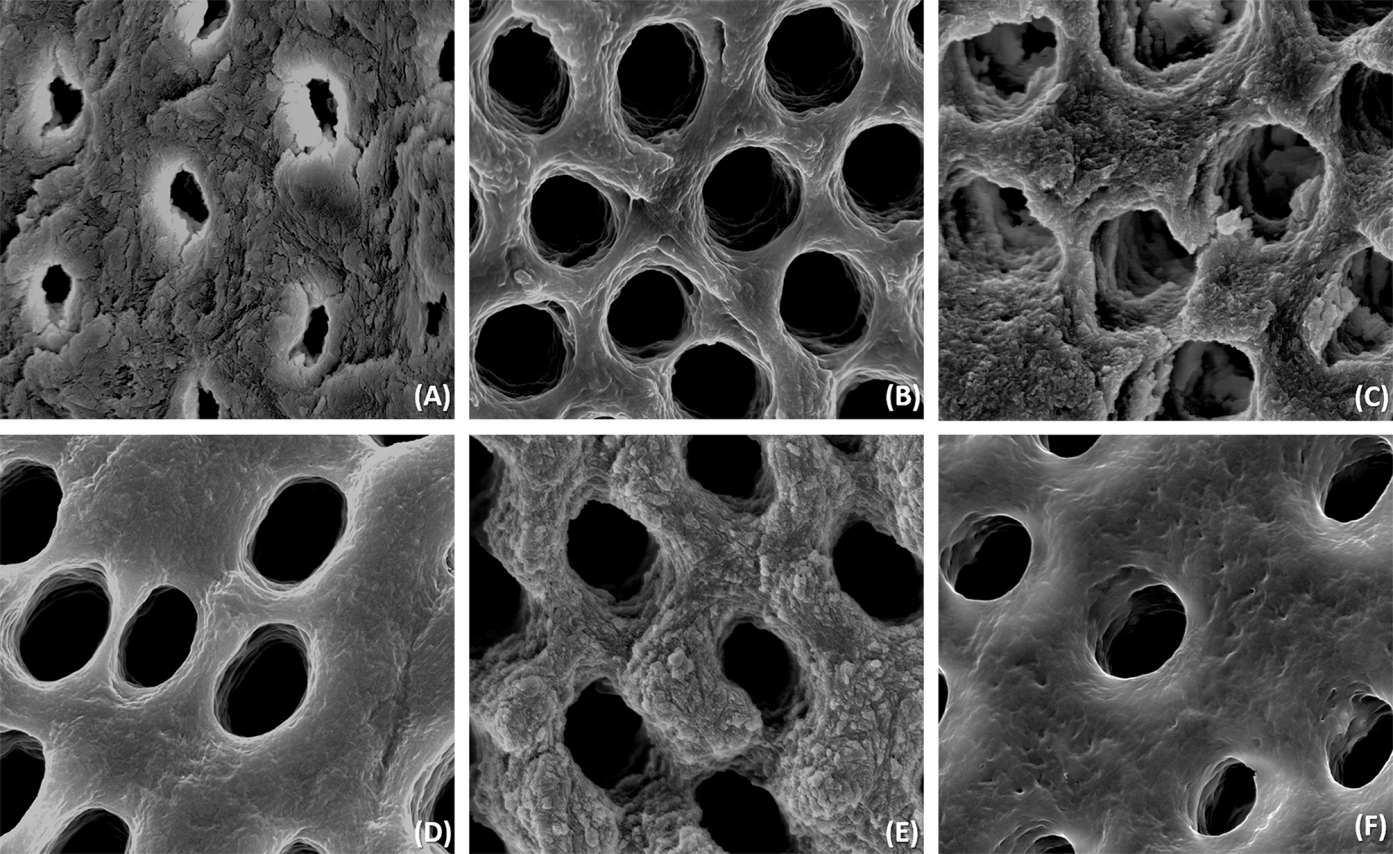
Scanning electron microscopic images of the dentin surface in the middle third of the root segments after treatment with the final irrigation protocols (25,000× magnification). (A) The organic components of the smear layer were removed by the 2.5% NaOCl, but the inorganic phase still partially covered the entrance of the dentin tubules; (B) The smear layer was completely removed by the use of 2.5% NaOCl followed by 17% EDTA, and the opening of the dentin tubules seems to be enlarged; (C) Erosion of peritubular and intertubular dentin with the dentin tubule orifices irregularly enlarged can be observed after the use of 2.5% NaOCl + 17% EDTA + 2.5% NaOCl; (D) Image of the group of 2.5% NaOCl + 17% EDTA + 2% CHX that presents a clean dentin surface very similar with the one observed in the group of 2.5% NaOCl + 17% EDTA; (E) A clean dentin surface was promoted by the use of the mixture of 5% NaOCl + 18% HEDP and the entrance of the dentin tubules were not enlarged by the use of these irrigants; (F) similar to the previous group, in the group of the mixture of 5% NaOCl + 10% Na_4_EDTA, the dentin surface is clean and the opening of the dentin tubules were not enlarged.

The SEM images taken from all groups showed that, except for G1, all irrigation protocols completely removed the smear layer produced by the root canal preparation ( Figure 1 ). In G2 and G4, it is possible to observe a discrete enlargement of the opening of the dentin tubules. In G3, the erosive effect of the irrigation sequence can be observed in the peritubular and intertubular dentin.

## Discussion

It is important to identify materials and techniques to be used during the endodontic treatment, to obtain stable BS values of the endodontic sealers to the root canal in a long term, since a good adhesion of the sealers to the dentin walls can reduce fluids percolation,^2^ bacterial re/contamination,^3^ and displacement of the obturation during a subsequent handling.^4 , 5^ This study results showed that the different irrigation protocols tested have a significant impact on the immediate and on the long-term dentin BS of AH Plus rejecting the null hypothesis. Although many studies evaluated the long-term stability of fibre-post bonding to dentin, the authors failed to find studies analyzing the effect of different irrigation protocols on the BS of endodontic sealers over time, except for two studies that presented the results after three and six months of the obturation.^28 , 29^

In this study, bovine teeth were used instead of human teeth since the dentin of the bovine incisor has a similar structure and number of tubuli to the human molar dentin.^30^ No differences were found between the bovine collagen and demineralized human dentin and from mineral matrices of human and bovine dentine.^31 , 32^ A study showed that the bovine dentin substrate did not influence the push-out BS of sealers compared to human dentin when using an intratooth model.^33^ Although bovine teeth can have dentin tubules larger than human teeth, the results of the groups are not skew since the push-out test can only rank the bonding of the root canal materials.^34^ Also, the samples from all groups can be more standardized than when human teeth are used, as they can be acquired from animals with similar age and stored for the same amount of time.

When root canals are filled with a core material, such as gutta-percha, and a sealer at least two interfaces can be dislodged from the canal space, this can create a systematic source of error.^34^ In this study, the root canals were filled only with the AH Plus sealer to have values of the BS truly between the dentin and the sealer. The complementary analysis of the failure patterns was not performed, since once the canals were filled only with the sealer, all failures had undoubtedly adhesive nature.^9 , 34^

The best results of initial BS were obtained in G2 (NaOCl + EDTA), G3 (NaOCl + EDTA + NaOCl), and G4 (NaOCl + EDTA + CHX). These groups were similar to each other and presented a clean surface with high roughness due to EDTA use,^6 , 13 , 35^ which favours the micromechanical interlocking of the endodontic sealers.^1^ The use of CHX after the decalcifying agent in G4 (NaOCl + EDTA + CHX) did not improve the initial BS when compared with G2 (NaOCl + EDTA), as observed in previous studies related to adhesion.^28 , 36^ In G2 (NaOCl + EDTA) and G4 (NaOCl + EDTA + CHX), the exposed collagen on the dentin surfaces probably contributed to the adhesion of AH Plus, since it was reported that the amino groups of the collagen fibers can bond chemically with this sealer.^14^ However, this contribution was not significant to produce statistical differences between these groups and G3 (NaOCl + EDTA + NaOCl), which had the collagen matrix deproteinated by the final irrigation with NaOCl.^7^ Another research with different sealers did not find differences in the BS with the use of NaOCl after the EDTA when compared with the group that applied EDTA as the last irrigant as well.^14^ These results reinforce the concept that the strong joints are most effectively created by the mechanical bonding.^1^

The specimens treated with the mixture of NaOCl and HEDP (G5) had initial BS similar to the groups with EDTA as the decalcifying agent (G2, G3, and G4). However, previous studies obtained higher values of BS for the mixture of NaOCl and HEDP compared to the conventional irrigation protocol of NaOCl followed by EDTA.^8 , 10 , 14^ A paper on FTIR analysis showed that after using the mixture of NaOCl and HEDP, more mineral than collagen can be found on the surface of the substrate.^7^ Consequently, the high push-out BS obtained in this group might have resulted from the cleanliness of the surface and reasons other than the interaction between collagen amino groups and AH Plus.^10^ Once the roughness of the dentin surface is not increased with the use of HEDP as much as when EDTA is used,^13^ an important factor to be considered is that HEDP is a bisphosphonate that strongly adsorb to hydroxyapatite surface and increase the surface-free energy.^37^ In the root dentin, this increase in surface-free energy results in high wettability of the canal walls,^13^ providing the necessary proximity between materials, facilitating molecular attraction. It results in a major penetration of the sealer into the irregularities of the surface, observed by the low gap distance between the filling material and the root dentin,^38^ and in an enhancement of the BS values due to the increase in the mechanical interlocking of endodontic sealers to dentin when HEDP is used to irrigate the root canals.^8 , 10^

Amongst the six irrigating protocols tested, G1 (NaOCl) was one of the groups that presented the lowest initial push-out strength value. This result agrees with previous studies that obtained lower values of BS for the group that used only NaOCl as the irrigant compared to groups that employed NaOCl associated with EDTA and/or HEDP.^8 , 10^ The possible reason for this outcome was the sealer adhesion to the inorganic components of the smear layer reducing the sealer resistance to the dislodgement forces.^8^ Since the mechanical bonding is the most effective mean to create a strong joint^1^ and NaOCl does not significantly increase the roughness of the dentin surface,^13^ this irrigation protocol does not favour a mechanical interlocking of the sealer to the dentin. Another reason that could be suggested for the low BS values is the oxidizing effect of the NaOCl that remains inside the root canal and negatively affects the BS of the resin-based sealers.^11 , 12^ However, this effect might have been insignificant in this study due to the final rinse with distilled water.^8^ Besides, in G3 (NaOCl + EDTA + NaOCl), the last irrigant used was also NaOCl and the push-out BS values of this group were significantly higher than the values of G1 (NaOCl).

The other group that presented the lowest initial push-out BS values was G6 (mixture of NaOCl + Na_4_EDTA). In this group, the smear layer is completely removed after the 5 min of use of the mixture, and the chemical composition of the surface is similar to a surface not treat with irrigants.^7^ However, no results were published regarding the effects of the mixture of NaOCl + Na_4_EDTA on the dentin roughness and, since this is the first study that tested the effects of this irrigation protocol on the BS of sealers to dentin, comparisons of these results with the literature were not possible.

After 20 months of aging, the rank of irrigation protocols from the highest to the lowest values of the push out BS was G3=G5>G6=G4>G1=G2. An important fact observed in the groups G3 (NaOCl + EDTA + NaOCl) and G5 (Mixture of NaOCl + HEDP) was the high occurrence of fractures of the dentin slices during the push-out tests.

When analysing the results of the same group over time, those with BS preserved (G3, G5, and G6) were smear layer free and did not present the collagen matrix exposed on the dentin.^7^ In G1 (NaOCl), the values of BS reduced after 20 months of the obturation when compared with the initial values, suggesting that the presence of smear layer on the surface can negatively affect the adhesion over time. Although G2 (NaOCl + EDTA) and G4 (NaOCl + EDTA + CHX) presented initial high BS values, a reduction occurred after 20 months of the obturation. The likely main reason for this find was a suboptimal infiltration of the sealer in the demineralized collagen matrix, followed by the degradation of the denuded collagen fibers by the action of metalloproteinases.^15^ G4 (NaOCl + EDTA + CHX) had a significantly better preservation of the BS than G2 (NaOCl + EDTA) after aging, agreeing with previous studies that reported some long-term stability of BS with the use of the CHX.^36 , 39^ Probably, in G4 (NaOCl + EDTA + CHX), the action of the metalloproteinases was partially suppressed by the protease inhibitor effect of CHX.^15 , 18^ However, the protective effects of CHX do not remain for long aging periods.^39^

This study did not have the intention to quantify the smear layer removal by the irrigation protocols tested since this is already well established in the literature, thus only one sample of each group was used to obtain the SEM images from the dentin surface. The photomicrographs obtained showed different characteristics on the dentin surface according to the irrigation protocols tested ( Figure 1 ). Since the NaOCl only affect the organic matter,^7^ the smear layer was not completely removed by this protocol and it is possible to observe inorganic matter covering the surface of the dentin on the photomicrography. The use of NaOCl associated with chelating solutions G2 (NaOCl + EDTA), G3 (NaOCl + EDTA + NaOCl), G4 (NaOCl + EDTA + CHX), G5 (mixture of NaOCl + HEDP), and G6 (mixture of NaOCl + Na_4_EDTA) promoted a complete removal of the smear layer in the samples of these groups. However, probably due to the higher demineralization capacity of 17% EDTA compared to Na_4_EDTA and HEDP^7 , 40^ , it is possible to observe an enlargement of the entrance of dentin tubules in G2 (NaOCl + EDTA), G3 (NaOCl + EDTA + NaOCl), and G4 (NaOCl + EDTA + CHX). In G3 (NaOCl + EDTA + NaOCl), the intercalated use of NaOCl and EDTA resulted in dentin erosion and irregular and rough tubules orifices, as previously described.^41^ In the SEM images, the microtopography appears to be rougher and irregular in G1 (NaOCl), G3 (NaOCl + EDTA + NaOCl), and G4 (mixture of NaOCl + HEDP). This feature might be the result of the higher amounts of mineral on the surface of these groups due to the collagen deproteination by the NaOCl,^7^ since the exposition of the collagen has been associated with smooth and plane images.^41^ However, this appearance is not related to major values of surface roughness.^13^

Within the limitations of this study, it was possible to rank the irrigation protocols tested that promote strong and stable dentin BS of AH Plus in a long term. The final irrigation protocols of NaOCl + EDTA + NaOCl and the mixture of NaOCl + HEDP resulted in high BS values for AH Plus to the dentin that were stable over time. These results seemed to be influenced by the alterations on the dentin surface characteristics promoted by the irrigation solutions. It reinforces the importance achieving clean surfaces that present high roughness and wettability to obtain good interfacial interaction between the adherend and the adhesive.^1^ However, more studies should be performed to understand the adhesive interface and the changes that occur in this region over time when different irrigation protocols and different types of endodontic sealers are used.

## Conclusions

Only the final irrigation protocols of NaOCl + EDTA + NaOCl and the mixture of NaOCl + HEDP resulted in high BS values for AH Plus to the dentin that were stable over time. These results seemed to be influenced by the alterations on the dentin surface characteristics promoted by the irrigation solutions.
